# Feasibility of FUS lung cancer treatment using unilateral lung flooding

**DOI:** 10.1186/2050-5736-2-S1-A21

**Published:** 2014-12-10

**Authors:** Frank Wolfram, Thomas G Lesser

**Affiliations:** 1 Department of Thoracic and Vascular Surgery, SRH Wald-Klinikum Gera, Teaching Hospital of Friedrich-Schiller-University Jena, Germany

## Background

Lung is not considered for HIFU ablation so far. By replacing air with saline, therapeutic ultrasound could penetrate parenchyma and reach underlying cancer. Unilateral lung flooding is known for ultrasound guided thoracoscopic surgery.

Lung cancer is prevalent in oncology and the most mortal cancer entity, its cure requires operability and results in loss of parenchyma. Whereby we were motivated to combine lung flooding and HIFU to create an alternative treatment option to surgery. Therefore the interaction between therapeutic ultrasound and cancer tissue needs to be investigated as well as the flooding process, to provide a sufficient time window for treatment.

## Materials & methods

Human lung lobes containing non-small-cell-lung-cancer (NSCLC) were flooded with saline following intra-surgical atelectasis. Ex-vivo ultrasound and MR images were acquired in state of gas free flooding. HIFU was applied into centrally located cancer tissue. The targeted cancer and lung tissue were histological analyzed using HE and NADPH staining. In vivo unilateral lung flooding was performed on three pigs (28-33Kg). Quality of flooding was assessed by ultrasound imaging, further vital parameters (HR, BGA- pO2, pCO2) were monitored. A stable flooding condition (1h) was maintained before re-ventilation.

## Results

In all animals, lung flooding was performed without remaining gas content. Parenchyma and surrounding mediastinal structures could be imaged by ultrasound through the lung. Vital parameters stayed inside physiological range (HR 45-91 bpm / pO2 (155-450mmHg)/ pCO2 (30-63mmHg)) through the procedures.

Lung tumors (NSCLC) could be well visualized by ultrasound and MR imaging with sharp delimitation to the surrounding parenchyma. Targeted lung cancer tissue appears slightly whitish with thorough areas of necrosis. NADPH stain confirmed complete ablation of lung cancer mass while surrounding parenchyma remains vital.

A summary of publications, describing the physiological impact of flooded lung and current trends in pulmonary oncology, will be presented.

## Conclusion

Ultrasound and MR guidance in flooded lung is feasible. HIFU passes flooded parenchyma and is absorbed by cancer tissue, which leads to its ablation. FUS using lung flooding is a potential treatment option for lung cancer and demands further research.

